# Supplementary material for: WordCommentsAnalyzer: A windows software tool for qualitative research
Source: F1000Res. 2018 Sep 4;7:536. Originally published 2018 May 3. [Version 2] doi: 10.12688/f1000research.14819.2 (PMC6137411; doi:10.12688/f1000research.14819.2)
Supplement: Supplementary file 3 [file f1000research-7-17614-s0002.tgz › b2a79496-4ada-47c4-9682-a99ce867836d.zip!/Eight Iranian Health Journals/Salmand_Iranian Journal of Ageing _9.docx]

© Salmand_Iranian Journal of Ageing . This work is licensed under CC BY-NC 4.0 (https://creativecommons.org/licenses/by-nc/4.0/).

Volume & Issue: دوره 11 - شماره 1

Title: مفهوم ارزیابی مراقبت در مراقبان خانوادگی سالمندان

http://salmandj.uswr.ac.ir/article-1-1074-fa.html

اهداف: با افزایش تعداد افراد سالمند، بیماری‌های همراه دوران سالمندی و به‌دنبال آن وابستگی و نیاز به مراقبت نیز درحال‌افزایش است که این امر اهمیت توجه به موضوع مراقبت خانوادگی و تجارب را دوچندان می‌کند. از بین مفاهیم مرتبط با مراقبت خانوادگی، مفهوم ارزیابی مراقب از کار خود نقش ویژه‌ای را در پذیرش، تداوم و پیامدهای مراقبت ایفا می‌کند. هدف از این مطالعه، مرور مفهوم ارزیابی مراقبت از کلیه جنبه‌های آن در مراقبان خانوادگی است. مواد و روش‌ها: مطالعه حاضر از نوع مروری سنتی یا حکایتی بود. به‌منظور مرور مفهوم ارزیابی مراقبت خانوادگی جستجوی رایانه‌ای کلیه مقالات بدون درنظرگرفتن محدودیت زمان از طریق پایگاه‌های اطلاعاتی انگلیسی‌زبان پروکوییست، گوگل‌اسکالر، پابمد و اسکوپوس با کلیدواژه‌های Appraisal, Caregiving, Family caregiving, Burden, Satisfaction, Positive aspect, Negative aspect, Older adults, Elderly و پایگاه‌های اطلاعاتی فارسی‌زبان مانند پایگاه اطلاعات علمی جهاد دانشگاهی (SID)، ایران‌داک و ایران‌مدکس با کلیدواژه‌های «سالمند، ارزیابی مراقبت، مراقبت خانوادگی، بار مراقبت و فشار مراقبت» انجام گرفت. یافته‌ها: ادراک مراقبان از مراقبت می‌تواند تحت‌تأثیر عوامل مختلف فردی، اجتماعی و فرهنگی قرار گیرد و همین ارزیابی ذهنی سبب می‌شود که با وجود استرس‌زابودن شرایط مراقبت، همه افراد درک یکسانی از شرایط محیط نداشته باشند و برخی تجربیات مثبتی در این شرایط داشته باشند و برخی دیگر استرس را بیشتر از آنچه وجود دارد درک نمایند که باعث ایجاد پیامدهای منفی برای سلامتی و رفاه مراقب می‌شود.  نتیجه‌گیری: سازه ارزیابی مراقبت، سازه‌ای چندبُعدی است و ابعاد مثبت و منفی دارد. با درنظرگرفتن همه‌جانبه این مفهوم می‌توان به درک بهتر و درنهایت، ارزیابی جامع‌تری از وضعیت مراقبان پرداخت و مداخلات مؤثر را برای ارتقای سلامت این عزیزان به‌کارگرفت.

Title: تاثیر هشت هفته تمرینات ترکیبی منتخب بر سطوح ایمنی هومورال و شاخص‌های هماتولوژیک مردان سالمند غیرفعال

http://salmandj.uswr.ac.ir/article-1-811-fa.html

اهداف: سالمندی با بی‌نظمی جنبه‌های عملکرد ایمنی همراه است و در این دوران با فرایند پیری پاسخ‌های ایمنی کاهش می‌یابد. به‌عبارت‌دیگر، با کاهش سلول‌های سیستم ایمنی -که توانایی شناسایی و حمله مستقیم به سلول آلوده را دارند-، پاسخ‌های ایمنی کاهش می‌یابد و در پی آن نقص ایمنی روی می‌دهد. ازآنجاکه فعالیت ورزشی می‌تواند بر عملکرد ایمنی تأثیر داشته باشد و از طرفی پیری با بی‌نظمی ایمنی پیش‌رونده همراه است، شناخت درباره اثرات ورزش بر عملکرد ایمنی در دوران سالمندی اهمیت می‌یابد. هدف از انجام این پژوهش، بررسی تأثیر هشت هفته تمرینات ترکیبی (هوازی-مقاومتی) بر غلظت ایمونوگلوبولین‌ها و کورتیزول سرمی مردان سالمند غیرفعال بود. مواد و روش‌ها: در این مطالعه نیمه‌تجربی، 24 مرد سالمند سالم با دامنه سنی 70-60 سال و نمایه توده بدنی 22-25 کیلوگرم بر مترمربع به روش نمونه‌گیری دردسترس و هدفمند انتخاب و به‌طورتصادفی ساده به دو گروه تجربی (12 نفر) و کنترل (12 نفر) تقسیم شدند. در این پژوهش گروه تجربی، برنامه تمرین ترکیبی را انجام دادند و گروه کنترل همان شیوه زندگی غیرفعال خود را ادامه دادند. برنامه تمرین ترکیبی (هوازی-مقاومتی) شامل: دویدن روی نوارگردان برای مدت هشت هفته، هر هفته سه جلسه به‌مدت 20 دقیقه دویدن روی نوارگردان با شدت 60 تا 70درصد حداکثر ضربان قلب ذخیره بود. برنامه تمرینات مقاومتی شامل 10 حرکت ایستگاهی به‌صورت دایره‌ای شامل: فلکشن ساق، اکستنشن ساق، پرس پا، اسکات، کشش زیر بغل، پرس سینه، حرکت صلیب با دمبل، جلو بازو، پشت بازو و حرکت پارویی با طناب بود که با بار اضافه معادل 70درصد یک تکرار بیشینه و 10 تکرار در دو وهله متوالی با زمان استراحت 30 ثانیه‌ای و دودقیقه‌ای بین هر ایستگاه و وهله صورت گرفت. در این پژوهش، نمونه‌های خونی 24 ساعت پیش از شروع تمرینات و 24 ساعت بعد از آخرین جلسه تمرین جمع‌آوری شد. مقادیر ایمونوگلوبولین‌های سرمی IgG ،IgM و IgA با دستگاه نفلومتری و کیت بیندینگ سایت و مقادیر کورتیزول سرمی به روش ایمونورادیومتریک با دستگاه گاماکانتر و کیت RIA اندازه‌گیری شد. برای برآورد اوج اکسیژن مصرفی، پروتکل ناختون روی دستگاه تردمیل مورداستفاده قرار گرفت. برای مقایسه میانگین‌های درون‌گروهی و بین‌گروهی، روش تحلیل واریانس اندازه‌های تکراری به‌کار گرفته شد و نتایج در سطح معنی‌داری 0/05>P آزمایش شدند. یافته‌ها: نتایج نشان داد برنامه تمرین ترکیبی 12/75درصدی غلظت ایمونوگلوبولین G سرمی سالمندان را کاهش داد (0/04=P). باوجود اینکه غلظت سرمی ایمونوگلوبولین M به‌واسطه تمرینات ترکیبی، 15/93درصد کاهش یافت، اما این کاهش از نظر آماری معنی‌دار نبود (0/31=P). علاوه‌براین، در سطح IgA سرمی سالمندان تغییر معنی‌داری مشاهده نشد (0/90=P). غلظت کورتیزول سرمی در گروه تجربی به‌میزان 8/33درصد افزایش معنی‌دار یافت (0/001=P). براساس نتایج، تغییرات میانگین‌های بین‌گروهی در متغیرهای IgG ، IgM و IgA، تفاوت معنی‌دار نداشت (0/05>P).  نتیجه‌گیری: انجام فعالیت‌های بدنی یکی از عوامل مؤثر بر تغییر روند کار سیستم دفاعی است که این امر به شدت، مدت، نوع تمرین و وضعیت آمادگی جسمانی، نوع تغذیه، حالات روحی و روانی و عوامل هورمونی بستگی دارد. براساس نتایج به‌دست‌آمده، تمرین شدید و طولانی‌مدت با افزایش روند کاتابولیکی ممکن است موجب تضعیف سیستم ایمنی شود.

Title: تأثیر دو شیوه تمرینی تکلیف دوگانه بر تعادل سالمندان: توانایی اختصاص توجه

http://salmandj.uswr.ac.ir/article-1-809-fa.html

اهداف: ناتوانی در اختصاص توجه به تعادل در تکالیف هم‌زمان، باعث زمین‌خوردن سالمندان می‌شود؛ بنابراین، هدف تحقیق حاضر مقایسه اثرات دو شیوه تمرینی تکلیف دوگانه بر تعادل افراد سالمند بود. مواد و روش‌ها: تحقیق حاضر از نوع تحقیقات نیمه‌تجربی بوده است. شرکت‌کنندگان این تحقیق را 36 نفر از سالمندان 65 سال و بالاتر واجد شرایط شهر کرمان که شرایط انتخاب و ورود به تحقیق را داشتند، تشکیل می‌دادند. افراد منتخب براساس نمره پیش‌آزمون برگ به‌طور تصادفی به 3 گروه 12 نفری، تقسیم شدند. گروه‌های تمرینی شامل گروه تمرینی تکلیف دوگانه با اولویت ثابت، گروه تمرینی تکلیف دوگانه با اولویت متغیر و گروه کنترل بودند. گروه تجربی تمرینات را به‌مدت 4 هفته و هفته‌ای 3 جلسه انجام دادند که مدت زمان هر جلسه تمرینی 45 دقیقه بود. تمرینات شامل سه دسته فعالیت‌های ایستادنی، انتقالی و راه‌رفتن بود که براساس دو اصل دشواری تکلیف حرکتی و ایمنی فرد و تقسیم‌بندی تکلیف حرکتی جنتایل انجام شد. برای بررسی تعادل در سالمندان، آزمون زمان راه‌رفتن و نشستن در دو شرایط تکلیف منفرد و دوگانه و نیز آزمون تعادلی برگ مورداستفاده قرار گرفت. برای تجزیه و تحلیل داده‌ها، از تحلیل کوواریانس، آزمون تعقیبی بونفرونی و نسخه 18 نرم‌افزار SPSS در سطح معنی‌داری 0/05≥P استفاده شد. یافته‌ها: نتایج تجزیه و تحلیل داده‌ها نشان داد در آزمون تعادلی عملکردی و آزمون زمان راه‌رفتن و نشستن در شرایط تکلیف منفرد، بین گروه کنترل و گروه تکلیف دوگانه با اولویت ثابت و متغیر تفاوت معناداری وجود داشت (0/05≥P)، به‌طوری‌که دو گروه تمرینی تکلیف دوگانه، عملکرد بهتری را نسبت به گروه کنترل نشان دادند.  علاوه‌براین، نتایج در مورد آزمون زمان راه رفتن و نشستن در شرایط تکلیف دوگانه نشان داد که بین گروه کنترل و گروه تکلیف دوگانه با اولویت ثابت و متغیر و همچنین بین دو گروه تمرینی تکلیف دوگانه با اولویت ثابت و متغیر تفاوت معناداری وجود داشت (0/05≥P). نتیجه‌گیری: با توجه به اینکه یافته‌ها نشان‌دهنده برتری تمرینات با تکلیف دوگانه به نسبت تمرینات منفرد و همچنین تمرینات تکلیف دوگانه با اولویت متغیر به نسبت تکلیف دوگانه با اولویت ثابت در شرایط تکلیف دوگانه است؛ بنابراین، به‌نظر می‌رسد علاوه‌بر نظریه ظرفیت محدود توجه که توجیه‌کننده افت عملکرد در نتیجه اجرای تکالیف هم‌زمان است، سازوکارهای دیگری نیز در این فرایند درگیر است که توانایی اختصاص و توجه در اجرای تکالیف به‌صورت هم‌زمان توجیه‌کننده این برتری و تفاوت است. ازاین‌رو می‌توان با طراحی تمرینات تعادلی مبتنی‌بر روش‌های تکلیف دوگانه، به‌ویژه تمریناتی مبتنی‌بر توانایی تغییر توجه، توانایی‌های شناختی و تقسیم مناسب توجه به این تکالیف را بهبود بخشید و با بهبود این توانایی‌ها خطر زمین‌خوردن را در این قشر از جامعه کاهش داد.

Title: تأثیر صدای سفید بر خواب بیماران سالمند بستری در واحد مراقبت کرونری

http://salmandj.uswr.ac.ir/article-1-901-fa.html

اهداف: اختلالات خواب در بیماران سالمند بستری در واحدهای مراقبت کرونری شیوع بالایی دارد و این اختلالات به‌همراه تغییرات وضعیت خواب مرتبط به سن در سالمند تشدید می‌شود. تحقیق حاضر با هدف تعیین تأثیر صدای سفید بر خواب بیماران سالمند بستری در واحد مراقبت کرونری انجام شد. مواد و روش‌ها: مطالعه حاضر، مطالعه‌ای نیمه‌تجربی بود که طی آن 40 بیمار سالمند بستری‌شده در واحد مراقبت کرونری که واجد شرایط ورود به مطالعه بودند، از طریق نمونه‌گیری دردسترس وارد مطالعه شدند. طی این پژوهش، کل مدت زمان خواب در شب و مدت زمان القای خواب بیماران قبل از مطالعه، در روز اول پذیرش و پس از یک هفته بستری در واحد مراقبت کرونری برای دو گروه بررسی شد. در مدت این یک هفته، برای گروه تجربه قبل از خواب به‌مدت دو ساعت صدای سفید (صدای امواج اقیانوس) پخش شد، اما گروه کنترل، مداخله‌ای دریافت نکرد. داده‌های پژوهش با آزمون‌های آماری (مجذور کی، تی زوجی و مستقل) با استفاده از نسخه 18 نرم‌افزار SPSS تجزیه‌و‌تحلیل شد. یافته‌ها: میانگین سنی شرکت‌کنندگان در گروه کنترل 6/19±68/75 سال و در گروه مداخله 5/15±67/05 بود. بیشتر شرکت‌کنندگان در این طرح سالمندان مرد (57/5درصد) بودند. بیشتر شرکت‌کنندگان با تشخیص سندرم حاد کرونری در واحد مراقبت کرونری بستری شده بودند (80درصد) و 65درصد از آنان سابقه بستری در بیمارستان را داشتند. در گروه کنترل میانگین مدت زمان خواب سالمندان در شب و بستری‌شدن در بیمارستان 0/86±7/61 ساعت بود که پس از مطالعه به 0/56±4/69 ساعت کاهش یافته بود. میانگین مدت‌زمان القای خواب از 5/4±20 دقیقه قبل از مطالعه به 5/4±28 دقیقه پس از مطالعه افزایش یافت که این یافته بیانگر افت معنی‌دار وضعیت خواب بود در بیماران سالمند پس از بستری‌شدن در واحد مراقبت کرونری بود (P<0/001). میانگین مدت زمان خواب شبانه قبل از مطالعه در گروه مداخله با گروه کنترل، تفاوت معنی‌داری را نشان نداد [0/91±6/75 (0/146=P)]، اما پس از مطالعه، میانگین مدت زمان خواب در گروه مداخله کاهش معنی‌داری را نشان نداد [0/91±7/04 (0/12=P)]. مدت زمان القای خواب بیماران سالمند در گروه کنترل نیز از 19/14±5 دقیقه قبل از مطالعه به 4/2±17 دقیقه کاهش یافت (0/17=P).مقایسه مدت زمان خواب شبانه و مدت زمان القا خواب دو گروه پس از مطالعه حاکی از تفاوت معنی دار دو گروه بود (P<0/001). نتیجه‌گیری: صدای سفید، مجموعه صداهایی با دامنه شدت صداهای متفاوت است. یافته‌های تحقیق حاضر نشان داد با وجود آنکه صدای سفید وضعیت خواب بیماران سالمند را از نظر آماری تغییر معناداری نداده است، اما از افت ساعت خواب و افزایش زمان القای خواب جلوگیری کرده است. استفاده از صدای سفید با توجه به افزایش آستانه تحریک شنوایی و اثرات القایی آن بر خواب و همچنین هزینه-اثربخشی مناسب آن، برای تعداد زیادی از بیماران سالمند بستری در واحدهای مراقبتی قابل‌استفاده است.

Title: بررسی قدرت باورهای فراشناختی معیوب برای پیش‌­بینی نگرش‌­های ناکارآمد در بیماران قلبی-عروقی سالمند و غیر­سالمند

http://salmandj.uswr.ac.ir/article-1-803-fa.html

اهداف: پژوهش حاضر با هدف تبیین نگرش­‌های ناکارآمد بیماران قلبی و عروقی بستری در بیمارستان از طریق باورهای فراشناختی معیوب به اجرا درآمد.  مواد و روش‌ها: این مطالعه مقطعی در سال 1391 در بیمارستان شریعتی اصفهان انجام شد. جامعه آماری پژوهش را بیماران قلبی-عروقی بستری در بیمارستان شریعتی اصفهان تشکیل دادند که از میان آنها 87 نفر بیمار قلبی-عروقی به روش تصادفی ساده انتخاب شدند. ابزارهای پژوهش را پرسشنامه باورهای فراشناختی­ معیوب (ولز و کارترایت-هاتن) و مقیاس نگرش­‌های ناکارآمد (وایزمن و بک) تشکیل می‌داد. داده‌‌ها با نسخه 18 نرم‌افزار SPSS و با استفاده از تحلیل رگرسیون چندگانه و ضریب همبستگی پیرسون تجزیه و تحلیل شد.  یافته‌ها: نتایج تحلیل رگرسیون نشان داد که به‌ترتیب نیاز به کنترل افکار دارای توان پیش‌­بین معنادار برای رضایت دیگران (0/05>P)، باورهای مثبت درباره نگرانی و کنترل­‌ناپذیری نگرانی دارای توان پیش­‌بین معنادار برای تمرکز بر خود (0/05>P)، باورهای مثبت درباره نگرانی دارای توان پیش­‌بین معنادار برای کمال­‌گرایی (0/01>P)، کنترل‌­ناپذیری نگرانی و اطمینان‌شناختی دارای توان پیش‌بین معنادار برای نیاز به تأیید دیگران (0/01>P) و باورهای مثبت درباره نگرانی دارای توان پیش­‌بین معنادار برای تمرکز بر موفقیت (0/05>P) هستند. نتیجه‌گیری: نتایج این پژوهش نشان داد که باورهای فراشناختی معیوب، توان پیش­‌بینی­‌کنندگی نگرش‌­های ناکارآمد را دارد که برای سلامتی بیماران قلبی-عروقی خطر­آفرین است.

Title: بررسی ارتباط پلی‌مورفیسم‌های rs1131445 و rs4072111 ژن مسیر التهابی اینترلوکین 16 با بیماری آلزایمر دیررس در ایران

http://salmandj.uswr.ac.ir/article-1-930-fa.html

اهداف: بیماری آلزایمر شایع‌ترین نوع زوال عقلی در سنین پیری است که بروز آن با افزایش سن بیشتر می‌شود. تحقیقات نشان داده است که سیتوکین­‌ها پروتیین‌­هایی هستند که در مسیر­های التهابی نقش ایفا می‌­کنند و میزان آن‌ها در بیماری‌های التهابی تغییر می­‌کند. بر مبنای این واقعیت که بیماری آلزایمر یک بیماری التهابی است، می‌­توان نتیجه گرفت که سیتوکین‌­ها قادرند بر بروز این بیماری تأثیرگذار باشند. IL-16، سیتوکینی است که نقش آن در پاره­‌ای از بیماری‌­های التهابی به اثبات رسیده است. این ژن یکی از ژن‌های کد‌کننده سیتوکین‌‌های مسیر التهابی است که می‌تواند در پاتولوژی التهابی مشاهده‌شده در اطراف پلاک‌های پیری ایجادشده در بیماری آلزایمر ایفای نقش کند و با پاتولوژی بیماری آلزایمر مرتبط باشد. براین‌اساس، هدف این مطالعه بررسی اثر دو پلی‌مورفیسم rs1131445 وrs4072111 از ژن IL-16 بر خطر ابتلا به بیماری آلزایمر دیررس در جمعیت ایرانی بود. مواد و روش‌ها: در این مطالعه 144 فرد مبتلا به بیماری آلزایمر که علایم ابتلا به آلزایمر آنها توسط روان‌پزشک و براساس معیارهای مصاحبه بالینی تأیید شده بود، به‌عنوان گروه بیمار و 173 فرد سالم به شرط نداشتن بیماری روانی به‌عنوان گروه کنترل انتخاب شدند. DNA افراد به روش سالتینگ‌اوت استخراج شد و واکنش PCR برای تکثیر قطعه موردنظر با استفاده از پرایمرهای طراحی‌شده در شرایط اپتیمایزشده برای هر پلی‌مورفیسم انجام گرفت. محصول PCR در ابتدا برای تأیید عملکرد صحیح PCR با استفاده از روش الکتروفورز ژل پلی‌اکریلامید (PAGE)‌ کنترل شد. سپس محصول PCR با روش RFLP  به کمک آنزیم محدودکننده موردنظر، بُرش داده شد و به کمک الکتروفورز ژل پلی‌اکریلامید، ژنوتیپ نمونه‌ها تعیین و ژنوتیپ افراد براساس روش PCR-RFLP مشخص شد. برای تجزیه‌وتحلیل آماری داده‌های به‌دست‌آمده از نسخه 2/3/1 نرم‌افزار OpenEpi و نسخه 11/5 نرم‌افزار SPSS استفاده شد. یافته‌ها: بررسی تحلیل آللی بین دو گروه جمعیت بیمار و سالم ضمن درنظرگرفتن فاصله اطمینان (%95=CI) و سطح معناداری 0/05 برای پلی‌مورفیسم rs1131445 نشان می‌دهد که رابطه آلل C با بیماری آلزایمر معنادار نبوده (0/656=P) و ژنوتیپ TC در مقایسه با ژنوتیپ TT، اختلاف معنادار آماری نشان نداده است (0/614=P). بااین‌حال بررسی تحلیل آللی برای پلی‌مورفیسم rs4072111، بین دو گروه جمعیت بیمار و سالم نشان می‌دهد که رابطه آلل T با بیماری معنادار است و این آلل در ایجاد بیماری نقش محافظتی دارد (0/008=P). علاو‌براین ژنوتیپ TC  به‌عنوان یک حالت محافظتی، رابطه معناداری را با بیماری نشان داد (0/007=P). نتیجه‌گیری: وجود پلی‌مورفیسم در برخی از ژن‌های مسیر التهابی، افراد را مستعد ابتلا به آلزایمر می‌کند. تغییرات ژنتیکی در توالی DNA از ژن IL-16  منجر به تغییر محصول سیتوکین یا تغییر فعالیت آنها می‌شود. معناداری ارتباط پلی‌مورفیسم rs4072111 و بیماری آلزایمر با پیش‌فرض این مطالعه مطابق بوده است و نقش احتمالی این پلی‌مورفیسم­ را در بیماری آلزایمر دیررس نشان می­‌دهد. با وجود این، نبود ارتباط میان پلی‌مورفیسم rs1131445 و بیماری آلزایمر دیررس با توجه به کوچک‌بودن حجم نمونه قابل اثبات نیست و برای اثبات آن به حجم نمونه بزرگ‌تری نیاز است.

Title: رابطه کیفیت خواب با افسردگی در افراد سالمند در سه ناحیه شهر تهران

http://salmandj.uswr.ac.ir/article-1-826-fa.html

اهداف: در دهه‌های اخیر به‌دلیل کاهش زادوولد، ارتقای بهداشت و اجرای برنامه‌های پیشگیرانه و افزایش امید به زندگی، جمعیت افراد سالمند در کشورهای گوناگون افزایش یافته است به‌گونه‌ای‌که از این پدیده، به‌عنوان خاکستری‌شدن جمعیت یا سالمندی اجباری یاد می‌شود. بررسی پیشینه موجود نشان می‌دهد بیشتر اوقات افراد سالمند با بیماری‌های جسمانی و مشکلات روانی گوناگونی مانند: مشکلات حرکتی، دردهای مزمن، اختلال در خواب، گوشه‌گیری و افسردگی روبه‌رو می‌شوند. ازاین‌رو، ضرورت دارد بخش عمده‌ای از منابع و خدمات بهداشتی و درمانی هر جامعه‌ای به افراد سالمند اختصاص یابد و به نیازها و مشکلات آنان بیشتر توجه ‌شود. ازاین‌رو، هدف از انجام پژوهش حاضر بررسی رابطه کیفیت خواب با میزان افسردگی در افراد مقیم‌ در خانه‌های سالمندان سه ناحیه شهر تهران در سال 1392 بود. مواد و روش‌ها: مطالعه حاضر از نوع طرح‌های توصیفی-تحلیلی بود که جامعه آماری آن را تمامی افراد مقیم خانه‌های سالمندان زیرپوشش سازمان بهزیستی استان تهران تشکیل می‌دادند. نمونه‌های موردمطالعه 90 نفر سالمند بودند که به روش نمونه‌گیری تصادفی خوشه‌ای با رعایت نسبت از سه ناحیه شمال، مرکز و جنوب‌ شهر تهران و با درنظرگرفتن میزان هوشیاری، توانایی پاسخ‌گویی و تمایل به شرکت در مطالعه انتخاب شدند. برای جمع‌آوری داده‌ها، از دو پرسش‌نامه‌ مقیاس کیفیت خواب پیتزبرگ و مقیاس افسردگی سالمندان استفاده شد و سپس داده‌های به‌دست‌آمده با روش آماری آزمون رگرسیون چندمتغیره مورد تجزیه‌وتحلیل قرار گرفت. یافته‌ها: 60درصد آزمودنی‌های این پژوهش را زنان سالمند تشکیل می‌داد. نتایج به‌دست‌آمده در خانه‌های سالمندان سه ناحیۀ شهر تهران به‌طوریکسان نشان داد که رابطه معناداری بین کیفیت خواب افراد سالمند با میزان افسردگی آنان وجود دارد. این رابطه در مورد کیفیت خواب به‌صورت معکوس (0/001>P) معنادار بود و با ابعاد اختلال‌های خواب، مصرف داروهای خواب‌آور و اختلال در عملکرد روزانه با افسردگی به‌طورمستقیم (0/001>P) رابطه معناداری را نشان داد. نتیجه‌گیری: یک ارتباط علّی دوطرفه بین افسردگی و اختلال در ‌خواب وجود دارد، به‌طوری‌که بی‌خوابی می‌تواند به افسردگی منجر شود و بالعکس. اگر نشانه‌های افسردگی درمان نشود، به‌مرور بر کیفیت خواب تأثیر منفی می‌گذارد و به‌طورکلی اختلال در ‌خواب با خلق افسرده و کیفیت زندگی پایین ارتباط متقابل دارد. ازاین‌رو، به‌منظور بالابردن کیفیت خواب و کاهش میزان افسردگی افراد مقیم خانه‌های سالمندان، لزوم برگزاری برنامه‌های مراقبت‌ ویژه در دوران سالمندی تأکید می‌شود. همچنین پیشنهاد می‌شود در پژوهش‌های آینده، ارتباط بین خواب، سبک زندگی و رفتار روزانه در افراد سالمند مورد بررسی قرار گیرد. از محدودیت‌های تحقیق حاضر می‌توان به عدم‌تعمیم نتایج به تمام جامعه سالمندان به‌دلیل محدودبودن مطالعه در سه ناحیه شهر تهران، اشاره کرد. همچنین به‌دلیل کنترل‌نکردن متغیرهایی مانند: تحصیلات و شرایط اقتصادی افراد سالمند، ممکن است تعمیم‌پذیری یافته‌ها محدود شود. درپایان، پیشنهاد می‌شود در پژوهش‌های آینده ارتباط بین کیفیت خواب، سبک زندگی و برنامه روزانه در خانه‌های افراد سالمند موردبررسی قرار گیرد.

Title: آیا افراد کهنسال از گذر زمان ادراک متفاوتی دارند؟: یک مطالعه مقایسه‌ای ازنظر سن و جنس

http://salmandj.uswr.ac.ir/article-1-802-fa.html

اهداف: ادراک زمان، مهارت شناختی بنیادی و پیچیده‌ای است که امکان ادراک زنجیره‌ای از رویدادها و فعالیت‌ها و پیش‌بینی وقوع حوادث در آینده را را فراهم می‌آورد. سرعت گذر زمان تحت‌تأثیر نحوه عملکرد مناطق مختلف مغز و فرایندهای شناختی پایه ازجمله توجه و حافظه قرار دارد. با توجه به تغییر کارکردهای مذکور در سنین مختلف هدف اصلی این پژوهش، تعیین تفاوت ادراک زمان در زنان و مردان جوان و کهنسال بود. مواد و روش‌ها: طرح پژوهش حاضر از نوع علّی-مقایسه‌ای بود و جامعه موردمطالعه را تمام زنان و مردان جوان (30-20 ساله) و کهنسال (70-60 ساله) ساکن شهرهای تبریز و مرند تشکیل می‌دادند. تعداد نمونه اولیه را 64 نفر دربرمی‌گرفت که در دو گروه 32 نفری (نصف زن و نصف مرد) قرار گرفتند. نمونه‌گیری این پژوهش به‌صورت نمونه‌گیری دردسترس صورت گرفت و در انجام پژوهش از ابزارهای اندازه‌گیری مانند معاینه مختصر وضعیت روانی، آزمون افسردگی بک و تکلیف بازتولید زمان استفاده شد. ابتدا تکلیف بازتولید زمان برای هریک از آزمودنی‌ها به‌صورت انفرادی به اجرا درآمد. اجرای آزمون افسردگی بک و آزمون معاینه مختصر وضعیت روانی به‌دلیل جلوگیری از ایجاد خستگی و تأثیر آن در درک زمان، بعد از اجرای تکلیف رایانه‌ای ادراک زمان صورت گرفت. در تکلیف بازتولید زمانی، تصویر دایره در صفحه‌نمایش رایانه در مدت زمان‌های 400، 500 و 600 میلی‌ثانیه به‌عنوان زمان کوتاه و 1800، 2000 و 2200 میلی‌ثانیه به‌عنوان زمان طولانی ارائه شد. درمجموع، تعداد 80 کوشش (40 کوشش برای بازه زمانی کوتاه و 40 کوشش برای بازه زمانی طولانی) به‌طورمتوالی و تصادفی ارائه شد. پس از ارائه هر محرک، آزمودنی با فشاردادن کلید فاصله صفحه‌کلید رایانه، مدت زمان حضور محرک را بازتولید می‌کرد. داده‌های 30 جوان (15 مرد و 15 زن) و 27 کهنسال (12 مرد و 15 زن) با استفاده از آزمون تحلیل واریانس چندمتغیری (مانوا) مورد تجزیه‌وتحلیل قرار گرفت. یافته‌ها: نتایج نشان داد که بین دو گروه سنی (جوان و سالمند) در متغیر ادراک زمان تفاوت معناداری وجود داشت (0/007>P)، به‌طوری‌که سالمندان در هردو بازه زمانی کوتاه و طولانی بازتولید کوتاه‌تری دارند، اما جوانان بازۀ زمانی کوتاه را طولانی‌تر و بازۀ زمانی طولانی را کوتاه‌تر از مدت زمان حضور محرک بازتولید کردند. این در حالی است که در متغیر جنسیت بین دو گروه (زن و مرد) تفاوت معناداری وجود نداشت (0/244>P). نتیجه‌گیری: یافته‌های این پژوهش نشان می‌دهد که سن بر ادراک زمان تأثیر می‌گذارد، به‌نحوی‌که زمان ادراک‌شده در نزد افراد کهنسال کوتاه‌تر از زمان فیزیکی است. بر مبنای الگوی دروازه توجه در تبیین ادراک زمان، چنانچه منابع توجه کمتری به گذر زمان اختصاص یا ظرفیت حافظه کاهش یابد، برآورد زمان کوتاه‌تر خواهد بود. براساس تغییرات شناختی مربوط به کهنسالی، با افزایش سن توانایی‌های شناختی افراد کهنسال تقلیل می‌یابد. ازآنجاکه شواهد نشان‌دهنده آن است که پردازش دقیق فواصل زمانی به ظرفیت حافظه و توجه بستگی دارد؛ بنابراین، تغییرات در هریک از فرایندهای شناختی مذکور می‌تواند در تفاوت‌های مربوط به سن در ادراک زمان تأثیرگذار باشد و احتمالاً به‌دلیل همین عوامل، بازتولید زمان در افراد مسن کوتاه‌تر می‌شود.

Title: رابطه کیفیت زندگی با علایم افسردگی در سالمندان

http://salmandj.uswr.ac.ir/article-1-797-fa.html

اهداف: باتوجه‌به روند روبه‌افزایش جمعیت سالمند در ایران، افسردگی کیفیت زندگی را در این گروه سنی به‌شدت کاهش می‌دهد و به‌دنبال آن، سبب انزواطلبی و دوری از جامعه و فعالیت‌های اجتماعی می‌شود. هدف از انجام این مطالعه تعیین رابطه کیفیت زندگی با علایم افسردگی در سالمندان بازنشسته شرکت صنعت نفت اهواز بود. مواد و روش‌ها: این پژوهش به‌صورت مقطعی و از نوع همبستگی بود و جامعه آماری آن را 200 نفر از سالمندان بازنشسته صنعت نفت اهواز که با استفاده از نمونه‌گیری تصادفی ساده انتخاب شدند، تشکیل می‌دادند. برای سنجش کیفیت زندگی پرسش‌نامه کیفیت زندگی (SF-12) و برای علایم افسردگی پرسش‌نامه افسردگی (GDS) به‌کاررفت و نتایج پژوهش به روش تحلیل واریانس و رگرسیون چندمتغیره و با استفاده از نسخه 16 نرم‌افزار SPSS مورد تجزیه‌وتحلیل قرار گرفت. یافته‌ها: براساس نتایج این مطالعه، سالمندان گروه سنی 65-61 سال بیشترین فراوانی را با حدود 46/5درصد و سالمندان گروه سنی 75-71 سال و 80-76 سال هریک کمترین فراوانی را با حدود 5 درصد نمونه به‌خود اختصاص داده بودند. در این مطالعه میانگین سنی نمونه‌ها 4±65 سال بود. سالمندان گروه‌های سنی مختلف از کیفیت زندگی و علائم افسردگی تقریباً یکسانی برخوردار بودند و از نظر هیچ‌یک از متغیرهای وابسته (کیفیت زندگی و علائم افسردگی) تفاوت معنی‌داری وجود نداشت (P<0/05). براساس یافته‌های پژوهش حاضر، رگرسیون پیش‌بینی علایم افسردگی سالمندان از روی مؤلفه‌های کیفیت زندگی (کیفیت جسمانی و کیفیت روانی) معنی‌دار است (0/05>P و 1/57=F). براساس نتایج مطالعه حاضر، تفاوت معنی‌داری ازنظر کیفیت زندگی در میان سالمندان بدون بیماری و سالمندان مبتلا به بیماری‌های دیابت، فشارخون و سرطان وجود داشت. همچنین، میان سالمندان مبتلا به دیابت و سالمندان مبتلا به بیماری سرطان، تفاوت معنی‌داری ازلحاظ کیفیت زندگی وجود داشت. نتیجه‌گیری: نتایج این پژوهش نشان داد در دوران سالمندی بین افسردگی و کیفیت زندگی ارتباط معنی‌داری وجود دارد؛ بنابراین، با توجه‌به نتایج مطالعه حاضر باید عوامل مرتبط با کیفیت زندگی سالمندان در مراقبت از این گروه مدنظر قرار گیرد. با ایجاد روابط آرامش‌بخش برای سالمندان و اشتغال آنان در فعالیت‌های سازنده، می‌توان اعتمادبه‌نفس را در آن‌ها افزایش داد. همچنین با تقویت امیدبه‌زندگی به آینده از ایجاد ناامیدی در سالمندان جلوگیری می‌شود. پیشگیری، کاهش عوامل ایجادکننده افسردگی و درمان افسردگی ممکن است باعث بهبود کیفیت زندگی افراد سالمند شود. به‌علاوه، انجام مطالعات بیشتر در آینده توصیه می‌گردد.

Title: تأثیر تکلیف دوگانه بر متغیرهای فضایی-زمانی راه‌رفتن سالمندان دارای ضعف تعادل

http://salmandj.uswr.ac.ir/article-1-970-fa.html

اهداف: مطالعه پارامترهای فضایی-زمانی و تغییرات آنها در گام‌برداری ناشی از تکلیف دوگانه، بررسی تقارن در عملکرد پای راست و چپ و مطالعه تغییرپذیری در این پارامترها کمتر موردتوجه محققان قرار گرفته است. هدف این پژوهش بررسی تغییرات رخ‌داده در پارامترهای فضایی-زمانی گام‌برداری به‌علت هم‌زمانی آن با تکلیف دوگانه شناختی در سالمندان مبتلا به ضعف تعادلی بود.  مواد و روش‌ها: داوطلبان شرکت در این پژوهش، شامل 30 سالمند مبتلا به ضعف تعادلی بودند که سن بالای 70 سال داشتند که از طریق آزمون‌های برگ و MMSE انتخاب شدند. افرادی که در آزمون برگ نمره پایین‌تر از 52 داشتند، در این مطالعه شرکت کردند و افرادی که در آزمون MMSE نمره پایین‌تر از 23 کسب کردند، از این مطالعه خارج شدند. پس از آشنایی با نحوه اندازه‌گیری و کسب رضایت‌نامه کتبی، شرکت‌کنندگان در دو وضعیت، راه‌رفتن طبیعی و راه‌رفتن با تکلیف دوگانه شناختی، یک مسیر 12 متری را طی کردند. پارامترهای فضایی-زمانی از طریق دوربین‌های ویکون ثبت شد. سپس با استفاده از نرم‌‌افزار پلی‌گن متغیرهای کادنس، سرعت راه‌رفتن، طول استپ، طول استراید، زمان استپ و استراید، زمان استنس و نوسان، زمان حمایت یک پا و حمایت دو پا، جداشدن پای مخالف، تماس پای مخالف با زمین و زمان جداشدن پنجه، برحسب درصدی از طول چرخه راه‌رفتن استخراج شد. تجزیه‌و‌تحلیل اطلاعات با روش اندازه‌گیری مکرر و با سطح معنی‌داری 0/05>P انجام شد.   یافته‌ها: نتایج نشان داد از میان یارامترهای موردبررسی، متغیرهای کادنس و سرعت راه‌رفتن در راه‌رفتن با اجرای تکلیف دوگانه نسبت به راه‌رفتن عادی کاهش معنی‌داری یافت. باوجوداین زمان استپ، زمان استراید، زمان حمایت دوگانه، زمان حمایت یک پا، مدت زمان استقرار و نوسان در راه‌رفتن با تکلیف دوگانه نسبت به راه‌رفتن عادی کاهش معنی‌داری نشان داد (0/05>P). شاخص عدم‌تقارن در راه‌رفتن همراه تکلیف دوگانه نسبت به راه‌رفتن عادی در پارامترهای کادنس، زمان استراید و طول استراید، افزایش معنی‌داری داشت و در بقیه پارامترها اختلاف معنی‌دار نبود. نتایج نشان داد تغییرپذیری پارامترهای گام‌برداری از یک گام به گام بعدی در راه‌رفتن با اجرای تکلیف دوگانه نسبت به راه‌رفتن عادی تغییر معنی‌داری نشان نداد.   نتیجه‌گیری: نتایج نشان داد در راه‌رفتن با اجرای تکلیف دوگانه نسبت به راه‌رفتن عادی، زمان اتکای دوگانه و زمان استقرار افزایش و نیز سرعت گام‌برداری کاهش می‌یابد که باتوجه‌به پیشینه تحقیقات، این عوامل با افزایش بی‌ثباتی و درنتیجه افزایش خطر سقوط ارتباط دارد. بنابراین در سالمندان مبتلا به ضعف تعادلی، اجرای تکلیف دوگانه هم‌زمان با راه‌رفتن می‌تواند خطر سقوط را افزایش دهد. باتوجه‌به افزایش عدم‌تقارن در راه‌رفتن با تکلیف دوگانه، به‌نظر می‌رسد هماهنگی و تقارن دوجانبه حساسیت زیادی به تکلیف شناختی هم‌زمان داشته باشد. این عدم‌تقارن در عملکرد بین پاها به‌عنوان خطر سقوط شناخته شده است. ازاین‌رو، باتوجه‌به نتایج بیان‌شده می‌توان نتیجه گرفت که راه‌رفتن در سالمندان مبتلا به ضعف تعادلی به عملکرد شناختی بیشتری نیاز دارد و انجام تکلیف هم‌زمان شناختی، موجب تداخل در منابع توجهی و درنتیجه تغییر در الگوی راه ‌فتن می‌شود. بنابراین پیشنهاد می‌شود که سالمندان مبتلا به ضعف تعادلی که با خطر سقوط بیشتری مواجه هستند، از توجه به تکالیف هم‌زمان شناختی در حین راه‌رفتن خودداری و فقط بر راه‌رفتن تمرکز کنند.

Title: مقایسه کیفیت زندگی سالمندان قبل و بعد از دریافت بسته خدمات توانبخشی روزانه سازمان بهزیستی

http://salmandj.uswr.ac.ir/article-1-1080-fa.html

اهداف: با افزایش جمعیت سالمندان توجه به مشکلات آنان در جامعه ابعاد تازه و گسترده‌ای یافته است. در این میان گروه‌های تخصصی توانبخشی در مراکز مراقبتی، روزانه تلاش می­‌کنند تا با ارائه خدمات به سالمندان تا جای ممکن از ورود آنان به مراکز اقامتی شبانه‌روزی جلوگیری کنند. سازمان بهزیستی بدین منظور بسته خدماتی خاصی را طراحی و آن را برای اجرا به مراکز ارائه‌دهنده خدمات ابلاغ کرده است. هدف کلی این مطالعه، مقایسه وضعیت کیفیت زندگی سالمندان قبل و بعد از دریافت بسته خدمات توانبخشی سازمان بهزیستی در مرکز روزانه توانبخشی کهریزک-محمدشهر کرج بود. مواد و روش‌ها: روش پژوهش حاضر، روش توصیفی-تحلیلی دوگروهی از نوع قبل و بعد بود و جامعه آماری آن را سالمندان مراجعه‌کننده به مراکز جامع توانبخشی تشکیل می‌دادند. در این پژوهش، 50 نفر از سالمندان مراجعه‌کننده به مرکز روزانه سالمندی کهریزک شماره 2 -واقع در محمدشهر کرج- در سال 94-1393 که معیارهای ورود به مطالعه را داشتند، به‌طورتصادفی در دو گروه 25 نفره مورد و شاهد قرار گرفتند. به‌منظور رعایت اخلاق پژوهش، افراد گروه شاهد در فهرست انتظار دریافت خدمات قرار داده شدند. ابزار پژوهش، پرسش‌نامه کیفیت زندگی (SF-36) و فرم جمع‌آوری اطلاعات جمعیت‌شناختی بود. کیفیت زندگی قبل، یک ماه و سه ماه بعد از شروع مطالعه در هر دو گروه اندازه‌گیری و برای تجزیه‌وتحلیل داده‌ها از نسخه 20 نرم‌افزار SPSS و آزمون تحلیل واریانس یک‌طرفه با اندازه‌گیری مکرر استفاده شد. یافته‌ها: در این مطالعه میانگین سنی دو گروه مورد و شاهد به‌ترتیب 43/66 و 68/1 بود. براساس آزمون تی، بین میانگین سن در دو گروه مورد و شاهد اختلاف معناداری وجود نداشت. علاوه‌بر‌این بین جنسیت، سطح تحصیلات و سابقه بستری نیز قبل از مطالعه اختلاف معناداری دیده نشد. یافته‌های مطالعه نشان داد در گروه مورد میانگین نمرات کیفیت زندگی در شروع مطالعه، یک و سه ماه بعد به‌ترتیب 43/95، 56/30 و 74/38 و در گروه شاهد به‌ترتیب 44/74، 49/79 و 57/82 بود. حداقل نمره‌ کسب‌شده سالمندان از پرسش‌نامه کیفیت زندگی 24/72 و حداکثر نمره کسب‌شده آنان از پرسش‌نامه کیفیت زندگی 86/11 بود. علاوه‌براین، یافته‌های پژوهش نشان داد که در شروع مطالعه، میانگین کیفیت زندگی در دو گروه مورد و شاهد اختلاف معناداری ندارد، اما ظرف یک و سه ماه بعد از مداخله، این اختلاف معنی‌دار شد (به‌ترتیب 0/015>P و 0/001>P). نتیجه‌گیری: براساس یافته‌ها، خدمات مراقبتی روزانه ارائه‌شده در مرکز کهریزک موجب افزایش معنادار کیفیت زندگی سالمندان شده است. بنابراین انتظار می‌رود این خدمات بتواند به‌عنوان شیوه‌ای مؤثر و مکمل در کنار مداخلات رایج دیگر و همچنین به‌عنوان مداخله‌ای غیردارویی در افزایش کیفیت زندگی وابسته به سلامت در سالمندان به‌کارگرفته شود براین‌اساس، می‌توان گفت که بسته خدماتی سازمان بهزیستی کارآیی لازم را برای مراکز روزانه در توانمندسازی سالمندان دارد.

Title: رابطه معناداری زندگی و حمایت اجتماعی ادراک‌شده در بین سالمندان ساکن آسایشگاه‌های شهر اصفهان

http://salmandj.uswr.ac.ir/article-1-969-fa.html

اهداف: هدف از پژوهش حاضر بررسی معناداری زندگی در بین سالمندان ساکن آسایشگاه‌های شهر اصفهان براساس میزان حمایت اجتماعی ادراک‌شده آنان از سه منبع خانواده، دوستان و افراد مهم زندگی‌شان بود.  مواد و روش‌ها: در این پژوهش، تعداد 120 نفر (68 نفر مرد و 52 نفر زن) از سالمندان زن و مرد ساکن آسایشگاه‌های شهر اصفهان از طریق نمونه‌گیری دردسترس انتخاب شدند و با استفاده از پرسش‌نامه‌های معناداری زندگی و حمایت اجتماعی ادراک‌شده زیمت و همکاران (1988)، مورد بررسی قرار گرفتند.  یافته‌ها: نتایج تحلیل رگرسیون گام‌به‌گام و چندمتغیری داده‌ها نشان داد که 28/2درصد تفاوت‌های فردی در معناداری زندگی سالمندان به تفاوت‌های فردی در مؤلفه‌های حمایت اجتماعی ادراک‌شده (خانواده، دوستان و افراد مهم) بین آنها مربوط است که به‌ترتیب عامل خانواده و افراد مهم، بیشترین تأثیر معنادار را در تبیین تغییرات و پیش‌بینی معناداری زندگی سالمندان داشته‌اند. این همبستگی از نظر آماری معنی‌دار است (0/001=P). همچنین مدل رگرسیونی تبیین‌شده نیز براساس آزمون تحلیل واریانس انجام‌شده، خطی و معنادار است؛ زیرا مقدار آزمون F برای تعیین معنی‌داری اثر متغیرهای مستقل بر معناداری زندگی سالمندان 15/353 با سطح معنی‌داری 0/001=P است. معنی‌داری ضرایب رگرسیون نشان می‌دهد خانواده به‌طورمعنی‌داری، معناداری زندگی سالمندان را پیش‌بینی می‌کند؛ یعنی 19/8درصد واریانس بین خانواده و معناداری زندگی سالمندان مشترک است که با اضافه‌شدن مؤلفه افراد مهم، شدت رابطه به 27/1درصد افزایش می‌یابد. نتیجه‌گیری: نتایج حاصل از این پژوهش نشان داد، سالمندانی که در سطح قوی‌تری از حمایت اجتماعی قرار دارند، احساس معنا در زندگی آنها نیز به‌طورمعناداری بیشتر است. به‌عبارت‌دیگر احساس معنا در زندگی، با سطوح حمایت اجتماعی ادراک‌شده قوی، ارتباط معناداری دارد و به‌گونه‌ای بخش مهمی از تفاوت‌های فردی در معناداری زندگی سالمندان، به تفاوت‌های فردی در میزان حمایت اجتماعی ادراک‌شده توسط آنان مربوط است؛ بنابراین، امروزه با توجه به دگرگونی‌های اجتماعی و ازدیاد مشغله فرزندان و کاهش ارتباط آن‌ها با والدین سالمند، پیش‌بینی می‌شود که کاهش حمایت درک‌شده از طرف خانواده توسط سالمندان -که مهم‌ترین مؤلفه پیش‌بینی‌کننده معناداری زندگی از میان دیگر مؤلفه‌های حمایت اجتماعی ادراک‌شده است-، میزان معناداری زندگی آنان را تحت‌تأثیر قرار دهد. با توجه به گفته‌های سالمندان در مصاحبه‌های انجام‌شده با آنان، نقش کل اعضای خانواده در افزایش کیفیت زندگی سالمندان و دستیابی به تجربه لذت‌بخش از مؤلفه‌های معناداری زندگی محسوب می‌شود. ازاین‌رو، انتظار می‌رود با افزایش کیفیت و کمیت ارتباط اعضای خانواده با سالمندان، بتوان حمایت موردنیاز آنان را از طریق خانواده تأمین کرد. این امر مستلزم آگاهی و درک کافی اعضای خانواده از شرایط جسمانی و روانی سالمندان است. این مطالعه نشان داد درمجموع با توجه به تأثیرگذاری حمایت اجتماعی ادراک‌شده سالمندان در معناداری زندگی آنان، توجه بیشتر به مؤلفه‌های حمایت اجتماعی ادراک‌شده در قشر آسیب‌پذیر سالمند احساس می‌شود.

Title: زمین‌خوردن و کنترل پاسچرال در سالمندان با عیوب انکساری چشم

http://salmandj.uswr.ac.ir/article-1-1012-fa.html

اهداف: عیوب انکساری چشم که شامل نزدیک‌بینی، دوربینی و آستیگماتیسم است، از مهم‌ترین اختلالات چشم در سرتاسر دنیا است. شواهدی وجود دارد که نشان می‌دهد اختلال بینایی می‌تواند منجر به زمین‌خوردن سالمندان شود. هدف از انجام این مطالعه، بررسی تأثیر اختلال بینایی حاصل از عیوب انکساری چشم بر شیوع زمین‌خوردن و کنترل پاسچرال سالمندان بود. مواد و روش‌ها: سالمندانی که برای معاینات چشم‌پزشکی به درمانگاه شهرداری منطقه 9 مراجعه کرده بودند، توسط همکاران اپتومتریست باتجربه تحقیق (با استفاده از چارت اسنلن و افتالموسکوپ) مورد معاینات چشم‌پزشکی قرار گرفتند. در صورت تمایل افراد برای شرکت در مطالعه پس از کسب موافقت آگاهانه، اطلاعاتی در مورد سن، جنسیت ابتلا یا عدم‌ابتلا به بیماری‌های چشمی خاص، تاریخچه جراحی چشم و سابقه زمین‌خوردن طی 12 ماه گذشته توسط پرسش‌نامه جمع‌آوری شد. براساس نتایج غربالگری بینایی انجام‌شده در این سالمندان 77 سالمند مبتلا به عیوب انکساری چشم شامل: 19 فرد دوربین (سن 5/85±67/47 سال)، 22 نفر آستیگمات (سن 5/60±70/32 سال) و 36 نفر مبتلا به آستیگماتیسم همراه دوربینی (سن 7/69±69/78 سال) و 23 سالمند (سن 29/29±70/5 سال) بدون هیچ اختلال بینایی خاص وارد مطالعه شدند. 70درصد شرکت‌کنندگان خانم و 30درصد مرد بودند که از نظر سابقه زمین‌خوردن و کنترل پاسچرال موردبررسی قرار گرفتند. برای بررسی کنترل پاسچرال از آزمون برخیز و برو (TUG) استفاده شد. از افراد خواسته می‌شد از صندلی بلند شوند، در یک مسیر 3 متری مستقیم راه روند، سپس بازگردند و دوباره روی صندلی بنشینند. زمان پیمودن این مسیر توسط افراد به ثانیه ثبت می‌شد. آزمون‌های آماری شامل: تحلیل واریانس برای مقایسه بین گروه‌ها از نظر کنترل پاسچرال و آزمون خی-اسکوئر برای مقایسه شیوع زمین‌خوردن هریک از گروه‌های مبتلا به عیوب انکساری نسبت به گروه کنترل سالم بود. یافته‌ها: از جامعه سالمندان موردبررسی در این تحقیق 23درصد (23=n) در گروه سالم، 19درصد (19=n) دچار دوربینی، 22درصد (22=n) مبتلا به آستیگماتیسم و 36درصد (36=n) دوربین با آستیگماتیسم بودند. 70درصد شرکت‌کنندگان خانم و 30درصد مرد بودند. شیوع زمین‌خوردن در گروه‌های سالم، دوربین، آستیگمات و دوربین با آستیگمات به‌ترتیب عبارت بود از: 15/8، 26/1، 22/7 و 38/9. با وجود اینکه شیوع زمین‌خوردن در افراد مبتلا به عیوب انکساری چشم نسبت به گروه کنترل بدون اختلال بینایی بالاتر نشان داده شد، اما نتایج آزمون خی-اسکوئر نشان داد بین گروه‌ها تفاوت معنی‌داری از نظر شیوع زمین‌خوردن وجود ندارد (0/25=P). همچنین نتایج تحلیل واریانس نیز نشان داد آزمون برخیز و برو (آزمون کنترل پاسچرال) نتوانست تفاوتی را بین گروه‌ها نشان دهد (0/64=P). نتیجه‌گیری: اختلال بینایی حاصل از عیوب انکساری چشم در سالمندان منجر به افزایش زمین‌خوردن آنها نشد. علاوه‌براین، نتایج آزمون TUG نتوانست اختلال تعادل را در این افراد نشان دهد؛ ازاین‌رو، مطالعات بیشتری در زمینه ارزیابی کنترل پاسچرال با دستگاه‌های پیشرفته‌تر و درنظرگرفتن عوامل خطرساز دیگر لازم است تا عوامل تعیین‌کننده در زمین‌خوردن سالمندان با عیوب انکساری چشم بهتر مشخص شود.

Title: ارتباط ناتوانی با افسردگی، وضعیت شناختی و روحیه در سالمندان

http://salmandj.uswr.ac.ir/article-1-960-fa.html

اهداف: هدف این مطالعه بررسی میزان ناتوانی و ارتباط آن با افسردگی، اختلال شناخت و روحیه در سالمندان بود. مواد و روش‌ها: در این مطالعه توصیفی-تحلیلی که در مرکز روزانه سالمندان مؤسسه خیریه کهریزک استان البرز (تنها مرکز این استان)  به روش نمونه‌گیری تمام‌شماری انجام شد، تعداد 90 نفر در مطالعه مشارکت کردند. برای بررسی ناتوانی، ابزار سنجش ناتوانی سازمان بهداشت جهانی نسخه دومپ به‌کارگرفته شد. این پرسشنامه ناتوانی فرد را در شش حوزه: برقراری ارتباط و درک مطلب، تحرک و جابه‌جایی، مراقبت از خود، سازش و زندگی با دیگران، وظایف مرتبط با زندگی روزمره و مشارکت اجتماعی موردبررسی قرار می‌دهد. وضعیت افسردگی با مقیاس افسردگی سالمندان، وضعیت شناختی با آزمون کوتاه شناختی و روحیه سالمندان با آزمون سنجش روحیه سالمندان فیلادلفیا مورد ارزیابی قرار گرفت. داده‌ها با استفاده از آزمون‌های خی-2، تی، من-ویتنی، کروسکال‌والیس و همبستگی اسپیرمن تجزیه و تحلیل شد.  یافته‌ها: میانگین نمره ناتوانی سالمندان 13/66±20/61 بود و بین زنان و مردان اختلاف معناداری (P=0/001) وجود داشت. نمره ناتوانی زنان بیشتر از مردان بود. در شش حوزه موردبررسی با ابزار سنجش ناتوانی سازمان بهداشت جهانی نسخه دومپ، بیشترین ناتوانی در حوزه‌های تحرک و جابه‌جایی، وظایف خانه و خانواده و مشارکت اجتماعی مشاهده شد. علاوه‌براین، میانگین نمره ناتوانی در حوزه‌های تحرک و جابه‌جایی (P=0/001)، وظایف خانه و خانواده (P=0/001) و مشارکت اجتماعی (P=0/005) در بین زنان و مردان اختلاف معناداری داشت؛ بدین‌معنا که نمره ناتوانی زنان بیشتر از مردان بود. در حوزه‌های برقراری ارتباط و درک مطلب، سازش و زندگی با دیگران و مراقبت از خود اختلاف معناداری بین مردان و زنان مشاهده نشد. همچنین میانگین نمرات ناتوانی در گروه‌های تحصیلی اختلاف معناداری نداشت. بین میانگین نمره ناتوانی و وضعیت افسردگی (P<0/001)، وضعیت شناختی (P<0/001) و روحیه سالمندان (P<0/001) رابطه معناداری وجود داشت. . این رابطه در افسردگی مستقیم و در وضعیت شناختی و روحیه، معکوس بود؛ یعنی با افزایش نمره افسردگی و کاهش نمره وضعیت شناختی و روحی،ه میزان ناتوانی سالمندان افزایش می‌یافت. بین ناتوانی و هر سه خر‌ه مقیاس روحیه، تهییج (P<0/001)، نگرش به پیری (P<0/001) و نارضایتی از تنهایی (P<0/001)، رابطه معنادار و معکوس بود. نتیجه‌گیری: ناتوانی سالمندان با افسردگی، وضعیت شناختی و روحیه آنان ارتباط معناداری دارد. بنابراین، با اقدامات پیشگیرانه و درمان به‌موقع در افسردگی، تقویت حافظه، به تأخیرانداختن اختلالات شناختی با اجرای برنامه‌های تقویت روحیه، ایجاد نگرش مثبت به پیری و افزایش رضایتمندی از زندگی در سالمندان، می‌توان میزان ناتوانی آنان را کاهش داد.

Title: مقایسه خودکارآمدی و احساس تنهایی در سالمندان ساکن و غیرساکن آسایشگاه سالمندی

http://salmandj.uswr.ac.ir/article-1-950-fa.html

اهداف: امروزه سالمندی به یک پدیده مهم جهانی تبدیل شده است و توجه به مسائل این مرحله یک ضرورت اجتماعی محسوب می‌شود. در این سن خودکارآمدی با ابعاد روانشناختی از جمله احساس تنهایی مرتبط است. سالم پیرشدن حق همه افراد بشر است و این امر بر اهمیت پدیده سالمندی و پیشگیری از مشکلات سالمندان و درنهایت ارائه بهتر مراقبت به این جمعیت آسیب‌پذیر می‌افزاید و ارتقای سطح خودکارآمدی و ابعاد روانشناختی سالمندان را از اهداف دست‌اندرکاران تأمین سلامت جامعه قرار می‌دهد. این مطالعه با هدف مقایسه خودکارآمدی و احساس تنهایی در سالمندان ساکن و غیرساکن آسایشگاه‌های سالمندی شهرکرد انجام شده است. مواد و روش‌ها: این مطالعه مقطعی از نوع توصیفی-مقایسه‌ای بود و نمونه‌های موردمطالعه را 100 نفر از زنان و مردان بالای 60 سال تشکیل می‌دادند. برای گردآوری داده‌ها از پرسش‌نامه سه‌بخشی شامل پرسش‌نامه خودکارآمدی شرر و پرسش‌نامه احساس تنهایی استفاده شد. داده‌ها پس از جمع‌آوری با استفاده از نسخه 16 نرم‌افزار SPSS مورد تجزیه‌وتحلیل قرار گرفت.  یافته‌ها: نتایج حاصل از داده‌ها نشان داد بیشتر شرکت‌کنندگان در هر دو گروه از نمونه‌های این پژوهش، زن 40 نفر (80درصد) بودند. میانگین سنی واحدهای ساکن در خانواده 8/22±75/58 سال و در آسایشگاه سالمندی 7/93±77/78 سال بود. همچنین با توجه به نتایج، میانگین و انحراف معیار خودکارآمدی در سالمندان ساکن خانواده (11/48±51/76) بیشتر از سالمندان ساکن آسایشگاه سالمندی (5/26±48/44) بود. براساس یافته‌ها خودکارآمدی با وضعیت اشتغال و رضایت از اعضای خانواده در سالمندان ساکن در خانواده ارتباط معنی‌داری داشت. همچنین بین خودکارآمدی با گروه سنی، محل سکونت، رضایت از سرای سالمندان و احساس تنهایی با میزان تحصیلات و رضایت از سرای سالمندان نیز ارتباط معنی‌داری وجود داشت. نتایج نشان داد میزان تنهایی در سالمندان ساکن آسایشگاه سالمندی (11/01±121/36) در مقایسه با میزان تنهایی در سالمندان ساکن خانواده (24/25±120/94) بیشتر است. همچنین در سالمندان ساکن خانواده بین خودکارآمدی و تمام ابعاد احساس تنهایی ارتباط معکوس معنیداری وجود دارد (0/001=P). نتیجه‌گیری: با توجه به پایین‌بودن میانگین نمره خودکارآمدی سالمندان ساکن سرای سالمندان، ضرورت توجه بیشتر مسئولان خانه‌های سالمندی به نیازهای سالمندان و آموزش‌های همگانی ترویج فرهنگ نگهداری از سالمندان در کانون خانواده، از مواردبسیار مهم و اساسی است. ازاین‌رو همه نهادها و سازمان‌هایی که اهداف مهمی همچون سالمند سالم را موردتوجه قرار می‌دهند، باید با برنامه‌ریزی، آموزش و مشاوره با خانواده‌های دارای سالمند، گامی مهم برای رسیدن به اهداف خود بردارند. همچنین با توجه به نتایج حاصل از ارتباط خودکارآمدی و احساس تنهایی، ارزیابی خودکارآمدی به‌عنوان یکی از موارد تأثیرگذار در ابعاد روانشناختی افراد درنظرگرفته شود؛ زیرا تقویت خودکارآمدی، موجب مدیریت استرس و بهبود سلامت روانی خواهد شد. با توجه به نتایج حاصل از ارتباط خودکارآمدی و احساس تنهایی، ارزیابی خودکارآمدی راهی برای حمایت از خودمراقبتی سالمندان محسوب می‌شود؛ چراکه تقویت خودکارآمدی موجب مدیریت استرس و بهبود سلامت روانی خواهد شد.

Title: روایی پایایی نسخه فارسی پرسش‌نامه سرمایه اجتماعی اونیکس در سالمندان

http://salmandj.uswr.ac.ir/article-1-746-fa.html

اهداف: برقراری روابط اجتماعی و ایجاد و تقویت شبکه اجتماعی در سالمندان ممکن است منجر به ایجاد سرمایه اجتماعی شود. هدف پژوهش حاضر تهیه نسخه فارسی پرسش‌نامه سرمایه اجتماعی اونیکس و بولن و ارزیابی میزان روایی و پایایی نسخه فارسی این پرسشنامه در سالمندان بود. مواد و روش‌ها: این مطالعه مقطعی روی 192سالمند بالای 60 سال انجام شده است. برای فرایند ترجمه و معادل‌سازی ابزار سرمایه اجتماعی، پروتکل پروژه بین‌المللی ابزار کیفیت زندگی به‌کاررفت. تأیید ویژگی‌های روان‌سنجی ابزار توسط پرسش‌نامه‌های افسردگی سالمندان (5 سؤالی)، سلامت اجتماعی و سلامت عمومی انجام شد و داده‌ها مورد تجزیه‌وتحلیل آماری قرار گرفت. یافته‌ها: در زمینه کیفیت ترجمه (وضوح ترجمه، زبان مشترک، یکسانی مفهومی و کیفیت کلی ترجمه) نتایج نشان داد که از دیدگاه مترجمان 3 و 4 بیشتر گزینه‌ها دارای وضوح ترجمه (86/6درصد)، کاربرد زبان مشترک (76/3درصد)، یکسانی مفهوم (76/3درصد) و کیفیت کلی ترجمه (78/9درصد) در حد مطلوب بوده است. همچنین از دید متخصصان در حوزه مناسب‌بودن ترجمه، پرسش‌نامه نسخه فارسی مطلوب در بقیه حوزه‌ها مطلوب و قابل‌قبول بوده و در هیچ‌یک از حوزه‌ها غیر‌قابل‌قبول نبوده است. ضریب همبستگی درون‌رده‌ای برای نمره خرده‌مقیاس‌ها بیش از 0/70 به‌دست آمد. هم‌خوانی درونی (ضریب آلفای کرونباخ) برای پرسشنامه 0/96 بوده است. در تحلیل عاملی که در این مطالعه روی سالمندان ایرانی انجام شد، 7 عامل به شکل منسجم شناسایی شدند که تفاوت‌های مشخصی با ساختاربندی اونیکس داشتند، به‌طوری‌که سؤالات مربوط به روابط همسایگی با گزینه‌های مشارکت اجتماعی در یک عامل قرار گرفت و گزینه‌های مربوط به کارآیی اجتماعی نیز بخشی در عامل مشارکت اجتماعی و برخی در عامل احساس اعتماد جای گرفت. این 7 عامل، درمجموع 48/71درصد واریانس نمره‌ها  را تبیین می‌کرد  (0/835=KMO). توزیع واریانس نمره‌ها برای هریک از 7 عامل به‌ترتیب عبارت بود از: مشارکت اجتماعی 58/11درصد، حس اعتماد 48/8درصد، ارزشمندبودن زندگی 44/8درصد، روابط کاری 83/6درصد، پذیرش تفاوت‌ها 79/6درصد، روابط خانوادگی 58/6درصد و انسجام محلی 83/6درصد. برای تعیین روایی هم‌زمان مقیاس آزمون افسردگی، سلامت اجتماعی و سلامت عمومی سالمندان بررسی شد که یافته‌ها نشان داد ارتباط منفی معناداری بین میزان افسردگی و سرمایه اجتماعی کل وجود دارد (0/001=P). همچنین ارتباط مثبت معناداری بین سطح سلامت اجتماعی و وضعیت سلامت عمومی با سرمایه اجتماعی کل (هر دو 0/001=P) مشاهده شد. نتیجه‌گیری: همان‌گونه که یافته‌ها نشان می‌دهد در مطالعه حاضر، فرایند معادل‌سازی پرسش‌نامه از کیفیت مطلوبی برخوردار است. نسخه فارسی این پرسش‌نامه در جامعه از روایی ظاهری (از نظر وضوح و سادگی و قابل‌فهم‌بودن هریک از سؤالات، پاسخ‌ها و توضیحات نسخه فارسی پرسش‌نامه سرمایه اجتماعی و مناسب‌بودن ترجمه پرسش‌نامه، مناسب‌بودن پرسش‌نامه برای جامعه ایران، قابل‌فهم‌بودن پرسش‌نامه و مناسب‌بودن پرسش‌نامه برای نیازسنجی)، روایی افتراقی (همخوانی درونی نسخه فارسی پرسش‌نامه)، پایایی در دفعات آزمون (نسبی و مطلق) و همخوانی درونی، در حد قابل‌قبولی است. ازاین‌رو، این ابزار برای ارزیابی سرمایه اجتماعی سالمندان مناسب به‌نظر می‌رسد.

Title: اثربخشی گروه‌درمانی شناختی-رفتاری بر راهبردهای مقابله با استرس در خویشاوندان مراقبت‌کننده مبتلایان به آلزایمر

http://salmandj.uswr.ac.ir/article-1-1083-fa.html

اهداف: آلزایمر یک بیماری پیش‌رونده و ازبین‌برنده مغز است که به‌طورجدی سبب آسیب به بخش تفکر و حافظه انسان می‌شود. این بیماری، شایع‌ترین نوع زوال عقل است که شامل مجموعه‌ای از علائم نظیر: ازدست‌رفتن حافظه، قضاوت و استدلال در افراد است که سبب تغییرات در خلق، رفتار و توانایی ارتباط می‌شود. مراقبت از مبتلایان به آلزایمر با رفتار و اعمالی که افراد مبتلا به آلزایمر انجام می‌دهند، در صورت بی‌کفایتی منابع کنارآمدن فرد، بیشتر برای خویشاوندان استرس‌زاست. گروه درمانی شناختی-رفتاری در آموزش به‌کارگرفتن راهبردهای مقابله‌ای سازگار، نقش مهمی داشته است. بنابراین، هدف این پژوهش بررسی اثربخشی گروه درمانی شناختی-رفتاری بر راهبردهای مقابله با استرس در خویشاوندان مراقبت‌کننده مبتلایان به آلزایمر بود.  مواد و روش‌ها: روش تحقیق حاضر نیمه‌آزمایشی و طرح آن پیش‌آزمون-پس‌آزمون و پیگیری با گروه کنترل بود. 32 مراقب داوطلب مراجعه‌کننده به انجمن آلزایمر ایران در سال 1390 به روش نمونه‌گیری دردسترس انتخاب و در دو گروه آزمایش و کنترل به روش تصادفی گمارده شدند. ابزار گردآوری داده‌ها پرسش‌نامه مقابله با استرس (CISS21) بود که پیش از آغاز گروه‌درمانی و در پایان دوره درمان در سه مرحله پیش‌آزمون، پس‌آزمون و پیگیری به‌وسیله گروه‌ها تکمیل شد. تجزیه و تحلیل داده‌ها با استفاده از تحلیل کوواریانس، تحلیل کواریانس چندمتغیره و اندازه‌گیری مکرر صورت گرفت. یافته‌ها: نتایج نشان داد مؤلفه‌های راهبردهای سازگار (مسئله‌مدار و سرگرمی اجتماعی) در اثر گروه درمانی شناختی-رفتاری در گروه آزمایش نسبت به پیش‌آزمون و گروه کنترل، به‌طورمعناداری افزایش یافته بود (0/001=P). این در حالی است که مؤلفه‌های راهبردهای ناسازگار (هیجان‌مدار و توجه گردانی) در اثر گروه درمانی شناختی-رفتاری در گروه آزمایش نسبت به پیش‌آزمون و گروه کنترل، به‌طورمعناداری کاهش یافته بود (0/001=P). نتایج طرح اندازه‌گیری مکرر بین 3 مرحله پیش‌آزمون، پس‌آزمون و پیگیری نشان داد که اثرات گروه درمان شناختی-رفتاری بر افزایش سبک مقابله‌ای سازگار (مسئله‌مدار و سرگرمی اجتماعی) و کاهش سبک مقابله‌ای ناسازگار (هیجان‌مدار و توجه گردانی) و نیز اثر تعامل بین گروه و تکرار معنادار بود و بین دو گروه آزمایش و گواه تفاوت معناداری وجود داشت. با توجه به نتایج به‌دست‌آمده، گروه درمانی شناختی-رفتاری توانست بر افزایش استفاده از راهبردهای مقابله با استرس سازگار و کاهش استفاده از راهبردهای مقابله با استرس ناسازگار در خویشاوندان مراقبت‌کننده مبتلایان به آلزایمر تأثیر معنادار داشته باشد و عامل زمان در کاهش اثربخشی گروه درمانی شناختی-رفتاری، از پس‌آزمون به پیگیری بی‌تأثیر بود. نتیجه‌گیری: براساس یافته‌های پژوهش حاضر، گروه درمانی شناختی-رفتاری می‌تواند استفاده از راهبردهای مقابله با استرس سازگار را افزایش و استفاده از راهبردهای مقابله با استرس ناسازگار را در خویشاوندان مراقبت‌کننده مبتلایان به آلزایمر کاهش دهد. این امر با عواملی مانند شناخت کامل بیماری آلزایمر و تأثیر آن بر روند زندگی، ایجاد فضایی برای ابراز وجود، ایجاد فضایی برای برقراری تعاملات اجتماعی، پی‌بردن به اهمیت ورزش و اختصاص زمان برای تفریح، آموختن تن‌آرامی در شرایط پراسترس، شیوه درک مسائل زندگی، مهارت حل‌مسئله، احساس کنترل، مدیریت زمان ارتباط دارد. با توجه به نتایج مثبت، استفاده از گروه درمانی شناختی-رفتاری به‌عنوان درمانی کم‌هزینه برای مراقبت‌کنندگان مبتلایان به آلزایمر و دیگر گروه‌های مراقبت‌کننده مبتلایان به بیماری‌های مزمن پیشنهاد می‌شود.

Title: تأثیر 8 هفته تمرین هوازی منتخب بر تعادل ایستا و پویای مردان سالمند سالم غیرفعال

http://salmandj.uswr.ac.ir/article-1-886-fa.html

اهداف: سالمندی به سال‌های نزدیک یا پیشی‌گرفته از میانگین طول عمر بشر اشاره دارد که پایان چرخه حیات انسان است. افراد سالمند قابلیت‌های احیاشونده محدودی دارند و بیش از دیگر بزرگسالان در معرض بیماری، سندرم‌ها و کسالت قرار دارند. زمین‌خوردن یکی از مشکلات گسترده دوران سالمندی است که در اثر ناتوانی در حفظ تعادل رخ می­‌دهد. این مطالعه با هدف بررسی تأثیر تمرینات هوازی بر تعادل ایستا و پویای مردان سالمند سالم غیرفعال انجام شد. مواد و روش‌ها: در این پژوهش نیمه‌تجربی، 26 مرد سالمند سالم آسایشگاه سالمندان شهر قزوین که داوطلب شرکت در پژوهش بودند به‌طورهدفمند انتخاب شدند و در دو گروه تجربی (13 نفر) و کنترل (13 نفر) قرار گرفتند. گروه تجربی به‌مدت 8 هفته و هر هفته سه جلسه در برنامه تمرین هوازی شرکت کردند. از پرسش‌نامه ثبت اطلاعات فردی برای کنترل دیگر معیارهای ورود استفاده شد که این معیارها شامل: استقلال در انجام کارهای روزمره، فقدان مشکل دید و نبود سابقه زمین‌خوردن در یک سال گذشته، نداشتن دررفتگی مفصلی یا مشکل آرتریت مزمن و همچنین نداشتن سرگیجه بود. در صورت داشتن مشکلات مذکور، شرکت‌کنندگان داوطلب از مطالعه حذف می‌شدند. تعادل ایستا و پویای آزمودنی­‌ها با استفاده از آزمون‌­های تعادلی لک‌لک و زمان برخاستن و رفتن در دو مرحله قبل و بعد از 8 هفته ارزیابی شدند. تجزیه و تحلیل آماری با استفاده از آزمون‌­های تی زوجی و تی مستقل انجام گرفت. یافته‌ها: نتایج آزمون تی مستقل اختالف معنی‌داری بین متغیرهای سن، قد و وزن دو گروه نشان نداد (P>0/05). ازآنجاکه برای تعادل ایستا از آزمون لک‌لک استفاده شد، میانگین زمان بیشتر بیانگر اثرگذاری مثبت بود، اما با توجه به اینکه پس از هشت هفته تمرین در تعادل ایستای گروه تجربی تغییر معناداری مشاهده نشد، شاید بتوان چنین نتیجه گرفت که عوامل مختلفی همچون عضلات ناحیه تحتانی از جمله نعلی، دوقلو و همسترینگ در حفظ تعادل ایستا نقش دارد و چون پروتکل تمرینی به‌کاررفته مربوط به تمرینات هوازی (پیاده‌روی و دویدن آهسته) بوده، باعث تقویت این عضلات نشده است. نتیجه‌گیری: به‌نظر می­‌رسد فعالیت هوازی منظم موجب بهبود تعادل پویا در سالمندان می‌­شود. در تعادل پویا، حرکت و راه‌رفتن نقش اساسی را ایفا می‌کند. برنامه تمرینی به‌کاررفته در پژوهش حاضر، شامل دویدن آهسته و پیاده‌روی بوده که تعادل پویا را بهبود بخشیده است. از دلایل احتمالی بهبود تعادل می‌توان به افزایش سازگاری‌های عصبی ناشی از تمرین، افزایش سطح آمادگی جسمانی و بهبود قدرت و دامنه حرکتی، بهبود وضعیت هوازی، زمان واکنش و وضعیت روانی، بهبود کنترل عصبی-عضلانی شامل کاهش تغییرپذیری در به‌کارگیری واحدهای حرکتی و بهبود هم‌زمانی واحدهای حرکتی اشاره کرد. همچنین در این مطالعه نشان داده شده است که بین ترکیب بدن و میزان فعالیت بدنی با تعادل ارتباط مثبت وجود دارد. بنابراین، تمرین هوازی به‌عنوان یک شیوه تمرینی مناسب برای افزایش تعادل توصیه می­‌شود.
